# Activation of Epithelial-Mesenchymal Transition and Altered β-Catenin Signaling in a Novel Indian Colorectal Carcinoma Cell Line

**DOI:** 10.3389/fonc.2019.00054

**Published:** 2019-02-15

**Authors:** Sanghamitra Mylavarapu, Harsh Kumar, Smita Kumari, L. S. Sravanthi, Misti Jain, Aninda Basu, Manjusha Biswas, Sivaram V. S. Mylavarapu, Asmita Das, Monideepa Roy

**Affiliations:** ^1^Invictus Oncology Pvt. Ltd., New Delhi, India; ^2^Department of Biotechnology, Delhi Technological University, New Delhi, India; ^3^Regional Centre for Biotechnology, Faridabad, India; ^4^School of Life Sciences, Manipal Academy of Higher Education, Manipal, India; ^5^India Innovation Research Center, New Delhi, India; ^6^Division of Cancer Biology, MITRARxDx India Pvt. Ltd., Bangalore, India; ^7^Department of Molecular Pathology, MITRARxDx India Pvt. Ltd., Bangalore, India

**Keywords:** colorectal cancer, signaling, EMT, β-catenin, mitotic defects

## Abstract

Colorectal cancer is the third major cause of cancer-related mortality worldwide. The upward trend in incidence and mortality rates, poor sensitivity to conventional therapies and a dearth of early diagnostic parameters pose a huge challenge in the management of colorectal cancer in India. Due to the high level of genetic diversity present in the Indian population, unraveling the genetic contributions toward pathogenesis is key for understanding the etiology of colorectal cancer and in reversing this trend. We have established a novel cell line, MBC02, from an Indian colorectal cancer patient and have carried out extensive molecular characterization to unravel the pathological alterations in this cell line. In-depth molecular analysis of MBC02 revealed suppression of E-cadherin expression, concomitant with overexpression of EMT related molecules, which manifested in the form of highly migratory and invasive cells. Loss of membrane-tethered E-cadherin released β-catenin from the adherens junction resulting in its cytoplasmic and nuclear accumulation and consequently, upregulation of *c-Myc*. MBC02 also showed dramatic transcriptional upregulation of β-catenin. Remarkably, we observed significantly elevated proteasome activity that perhaps co-evolved to compensate for the unnaturally high mRNA level of β-catenin to regulate the increased protein load. In addition, there was substantial misregulation of other clinically relevant signaling pathways that have clinical relevance in the pathogenesis of colorectal cancer. Our findings pave the way toward understanding the molecular differences that could define pathogenesis in cancers originating in the Indian population.

## Introduction

Colorectal cancer (CRC) ranks as the third most commonly diagnosed disease and a leading cause of cancer-related mortality worldwide. It is further complicated by the fact that about 50% of CRC patients develop liver metastases during their lifetime (1). Although there has been a significant advancement in the development of treatment regimens, there is no effective therapy in the clinic for advanced CRC presenting with metastasis. Recently, shifting trends have emerged in the global incidence of CRC whereby rapidly developing countries present an increase in both CRC related incidence and mortality. In the developed nations however, there is an upward trend in incidence whereas mortality rates are either stabilizing or decreasing over time (1). CRC in India differs from the trends described in the developed nations mainly due to limited healthcare access coupled with poor socioeconomic backgrounds. In the Indian subcontinent, the annual incidence of colon and rectal cancers are 4.4 and 4.1 per 100,000 respectively (2). Additionally, there are reports of younger patients being usually diagnosed at an advanced stage (3). In recent years, understanding the underlying mechanisms that govern pathogenesis and progression of CRC have been the focus of research. However, majority of studies have been conducted on tumors and cell lines derived from Caucasian patients residing in developed countries. Consequently, most therapeutics used in the clinical management of CRC are based on data generated from the Caucasian population that often prove suboptimal for Indian patients. Since Indian population is genetically diverse (4), defining the contribution of genetic factors that lead to disease progression becomes even more critical for the management of Indian CRC patients.

Molecular alterations are the key contributors toward pathogenesis and progression of CRC, characterized by clearly defined stages starting from early adenoma, intermediate adenoma, late adenoma and carcinoma, leading into the final stage of cancer metastasis (5). Although germline mutations play a predominant role in many CRC patients, the vast majority of CRC cases are sporadic with no prior family history (6). Sporadic pre-cancerous polyps or lesions accumulate further genetic and epigenetic aberrations over time resulting in uncontrolled cellular growth, subsequently enabling the cells to acquire invasive and metastatic properties (7). An insight into the molecular events driving these cellular transitions, particularly toward enhanced invasiveness and metastasis, is crucial for the development of novel treatment regimens to combat CRC. One such critical event is the epithelial to mesenchymal transition (EMT), a series of programmed molecular and biochemical changes that result in cells losing their epithelial features while gaining mesenchymal features, such as loss of cell-cell contact, attaining an elongated spindle shaped morphology and increased motility. Complex networks of signaling pathways orchestrate this phenomenon. The inducers of EMT can downregulate E-cadherin while enhancing the N-cadherin and vimentin levels through modulating EMT-related signaling pathways, including Wnt/β-catenin, TGF-β, and EMT transcription factors, namely, zinc finger E-box binding homeobox (Zeb1/2) and Snail (8). This results in pathological changes in the tissue of origin, marked by uncontrolled cellular proliferation that eventually enables them to form secondary metastatic tumors (9). Activation of the EMT program is contextual whereby the incoming signals from the tumor microenvironment drive the molecular changes that ultimately set in motion the transition of cells from epithelial to mesenchymal states (10, 11). Among the myriad signaling molecules and pathways that are involved in the EMT program, crosstalk between these signaling pathways contributes toward pathogenesis and progression of CRC (12–14).

One of the key pathways involved in the EMT transition is the evolutionarily conserved Wnt/β-catenin signaling pathway that is involved in tissue morphogenesis during embryonic development (15, 16). β-catenin, the central effector in this pathway, participates in numerous cellular processes that are spatio-temporally separated. At cell-cell adherens junctions, β-catenin binds directly to the membrane anchored E-cadherin to form a catenin-cadherin complex—a critical mechanism that regulates localized cytoskeleton modulation (16–18). This interaction is thought to be a protective mechanism against proteasomal degradation, stabilizing β-catenin at the adherens junction (19, 20). Loss of E-cadherin from the membrane releases free β-catenin into the cytoplasm that eventually translocates into the nucleus and acts a transcriptional co-activator of pro-growth genes. Suppression of E-cadherin expression has been implicated in progression of cancer and is associated with poor prognosis and poor survival in many malignancies, including CRC (21). In the absence of an incoming Wnt signal, phosphorylation of a cluster of serine, and threonine residues at the N-terminus of β-catenin primes it for ubiquitination followed by proteasomal degradation, thus maintaining low levels of β-catenin in the cell. Mutations in these residues prevent phosphorylation resulting in stabilization and accumulation of β-catenin and subsequent increase in β-catenin mediated transcriptional activity (22–25). This is a tightly regulated step during normal cellular function—mis-regulation due to pathological transformation may lead to over-activation of pro-growth genes leading to abnormal cellular proliferation, a hallmark of cancer (26, 27).

In this article, we describe a novel CRC cell line, MBC02, derived from a patient of Indian origin that exhibited features of having undergone at least partial EMT. Significant overexpression of Wnt-β-catenin, TGFβ, and Notch pathways indicated an overall misregulation of clinically relevant signaling pathways that are also implicated in EMT. Transcriptional suppression of E-cadherin along with increased expression of N-cadherin, vimentin and EMT-related transcription factors, Twist and Zeb were corroborated by phenotypic changes such as enhanced migration and invasiveness and reduced response to standard-of-care therapeutics. MBC02 showed transcriptional upregulation along with cytoplasmic and nuclear accumulation of β-catenin, indicating activation of the Wnt/β-catenin signaling pathway in this cell line. In addition, increased nuclear accumulation of β-catenin could be linked to elevated levels of Pin1 expression in MBC02. These observations were further correlated with phenotypes that are typical of β-catenin driven mitotic defects such as supernumerary centrosomes at interphase and multipolar spindles at metaphase, resulting in cytokinetic failure and an enrichment of multinucleate cells that are aneuploid.

## Materials and Methods

### Immunohistochemical Analysis of Primary Tumor Sections

Immunohistochemical staining was performed on sections prepared from paraffin-embedded tissue blocks. Sections of ~3 μm thickness were collected on positively charged slides (TOMO IHC adhesive glass slides, TOM-11). The slides containing the tissue sections were heated at 60°C for 1 h, followed by deparaffinization and rehydration by passing through gradient of ethanol solutions and finally placed in deionized water. Heat induced antigen retrieval was performed using citrate based antigen unmasking solution (Vector labs, Cat. No. H3300). Endogenous peroxidase was blocked by incubating the tissue sections in 6 ml of 30% hydrogen peroxidase solution for 20 min. Ten percent normal goat serum (Vector lab Cat. No. S-1000) was used for blocking non-specific proteins prior to incubation with primary antibody for 1 h, followed by washing with 1X PBS. The tissue sections were incubated with primary antibodies for 1 h, washed with 1X PBS and then incubated with HRP labeled secondary antibodies. The sections were again washed with 1X PBS. Freshly prepared chromogenic reagent (anti-rabbit HRP, Cell Signaling, Cat. No. 8114s or anti-mouse HRP, Cell Signaling, Cat. No. 8125s) was added to the sections for developing the color to aid visualization of staining. Dako Envision Kit (Cat. No. K5007) was used for Ki-67. All slides were counter-stained with hematoxylin (Merck, Cat. No. 6092530121730), dehydrated by passing through gradient of ethanol solutions, placed in xylene and finally mounted and sealed. Representative images were captured in 200X magnification using Leica's Aperio ImageScope software (V12.3.3.5048).

### Establishment of MBC02

Surgically removed tumor tissue was obtained from the primary tumor site of a 37-year-old Indian female CRC patient with disease staging at T_2_N_1_M_0_, after obtaining informed consent under Institutional Review Board (IRB) approved protocol of MITRARxDx India Pvt. Ltd., India (IRB# TS-04-2011). The tumor tissue was washed extensively with 1X PBS, followed by incubation with 1X penicillin/ streptomycin for 10 min at room temperature. The tumor tissue was then sectioned to obtain 0.5–2.0 mm^3^ size sections. These were then digested with 0.5X collagenase in DMEM containing 1X penicillin/streptomycin at 37°C for 2–4 h. The disaggregated tumors were passed through a cell strainer and centrifuged to obtain a cell pellet. The cells were then counted by trypan blue exclusion method and seeded in flasks. Unattached cells and tumor debris were removed by changing the media, followed by differential trypsinization to remove fibroblasts. Further inhibition of fibroblast was achieved by growing the cultures in low serum and calcium containing media. The cultures were treated with 0.01% EDTA for 3 min and replaced with media containing 5% FBS for 24 h. Detached fibroblasts were removed by replacing with fresh media. This procedure was repeated for three passages to enrich the cultures for tumor cells. MBC02 cell line was established after sequential passaging and subsequent growth in phenol red free Dulbecco's Modified Eagle Medium (DMEM) containing 5.5 mM glucose supplemented with 10% FBS and 1X gentamicin. All assays reported in this article were carried out using cells between passages 6 to 20.

### Cell Culture

MBC02 cells were grown and maintained in phenol red free DMEM—low glucose (HiMedia, Cat. No. AL183A) medium, containing 5.5 mM glucose, 10% fetal bovine serum (FBS) and supplemented with 1X gentamicin. HCT116, HT29, and SW620 cells were obtained from the American Type Culture Collection (ATCC), grown and maintained in DMEM—high glucose (HiMedia, Cat. No. AL007A) containing 25 mM glucose, supplemented with 10% FBS and 1X penicillin and streptomycin. All cell lines were grown at 37°C, 5% CO_2_ atmosphere with relative humidity of 95%. Growth characteristics of MBC02 cells were measured by seeding cells at an initial concentration of 15,000 per well of a 12 well dish and allowed to grow. Cells were trypsinised from a single well and counted at designated time intervals. The population doubling time was calculated using the following formula: DT = T Log2/Log (X_e_/X_b_), where, T is the time period, X_e_ is the number of cells at the end of the incubation time and X_b_ is the number of cells at the beginning of the incubation time.

### Karyotype Analysis

MBC02 cells from passage number 8 and 16 were cultured in 5 ml of recommended media supplemented with 10% FBS and incubated in a humidified CO_2_ incubator at 37°C. Demecolcine solution (Sigma Aldrich, Cat. No. 7385) was added at a final concentration of 100 ng/ml and the cells were incubated for various time intervals (overnight, 24 and 48 h). Cells were harvested and centrifuged at 1,200–1,500 rpm for 10 min at room temperature. The cell pellets were resuspended in 8–10 ml of warm hypotonic solution (0.075 M KCl) and incubated at 37°C for 20 min, followed by addition of 500 μl of pre-cooled fixative (3:1 solution of methanol and acetic acid). The cells were gently mixed for uniform fixation. The cells were centrifuged and resuspended in fresh fixative. This process was repeated twice. The resuspended cells were placed in −20°C overnight. Next day, the fixed cells were dropped onto freshly washed glass slides and allowed to air dry. The dried slides were baked at 65°C for 8–12 h. The slides were then sequentially rinsed with trypsin solution (6.25 mg of trypsin in 50 ml Gurr buffer), followed by normal saline and deionized water. The washed slides were stained using Giemsa solution (2.0 ml of Giemsa stain added to 48 ml of Gurr buffer) for 5–6 min and washed with deionized water. Analyses of the stained slides were carried out using Cytovision software (Leica Biosystems).

### Mutational Analysis

Genomic DNA was extracted from tumor tissues and MBC02 cell line using a QIAamp DNA Micro Kit (Qiagen) and subjected to PCR using region-specific primers to detect the mutational status of *KRAS* (codon numbers 12, 13, 61, and 146) and *BRAF* (codon 600). DNA fragment containing *KRAS* mutation hotspots were amplified with the intron-based primers (28). Reaction mix contained 2.5 mM MgCl_2_, 0.2 mM dNTPs, 1 μM of each primer set, and 0.5 units of PhusionTaq (ThermoFisher Scientific) in a total volume of 50 μl. SW480 bearing mutation in *KRAS* and Caco2 harboring wild type *KRAS* were used as controls for PCR and sequencing reactions. PCR was carried out at 95 °C for 5 min, followed by 25 cycles at 95 °C for 30 s; 60 °C for 30 s and 72 °C for 30 s with a final extension for 5 min. PCR products were resolved on 1.5% agarose gel. The amplicons were excised and purified using a QIAquick gel extraction kit according to manufacturer's protocol (Qiagen) and processed for Sanger sequencing.

### Anchorage Independent Growth Assay

Tumorigenic potential of MBC02 cells was asessed using the anchorage independent growth assay. The base layer of agar (0.5%) was prepared by mixing 9 ml of complete media to 1 ml of 5% agar. The temperature of the solution was maintained at 50°C to prevent premature solidification of the agar. 1 ml of the agar mix was added to each well of a 6 well plate and allowed to solidify completely. The cells were washed with 1X PBS and harvested by trypsinization. The cells were centrifuged and resuspended in 1X PBS and counted. The cell number was adjusted to 5 × 10^3^cells/ml in complete media. The top agar layer (0.3%) was prepared by adding 0.6 ml of 5% agar to 9.4 ml of complete media containing cells. 1 ml of the top agar was layered over the base agar and allowed to solidify completely. 800 μl of complete media was layered on top to prevent drying of the agar. The plates were incubated at 37°C, 5% CO_2_ atmosphere with relative humidity of 95% for 2 weeks. Colonies were imaged using Nikon TiE inverted microscope.

### Cell Cycle Analysis

The culture media was removed and cells were washed with 1X PBS. Cells were harvested by trypsinization and collected by centrifugation at 2,000 rpm for 5 min. The cell pellets were washed twice with PBS and centrifuged at 2,000 rpm. The cells were resuspended in 1 ml PBS to obtain single cell suspension and fixed in ice cold 70% ethanol for at least 4 h at 4°C. After fixation, the ethanol was removed by centrifugation and the cells were washed twice with 1X PBS. Staining solution was prepared by adding propidium iodide at a final concentration of 50 μg/ml and RNAse A at a final concentration of 50 μg/ml. The samples were incubated at 37°C for 20 min and data acquired by flow cytometry (BD FACS Verse). Three biological replicates were performed to obtain statistically significant data.

### Cell Migration and Invasion Assay

For would healing assay, MBC02 and HCT116 cells were seeded in 6 well plates and allowed to grow to confluency. After generating a wound in the monolayer, the media was removed and the cells were washed to remove detached cells. The cells were fed with fresh media and the wound was allowed to close. The gap between the invasion fronts was measured at regular interval to calculate the rate of wound closure. We used the transwell migration assay to evaluate the migratory and invasive potential of MBC02 in comparison to HCT116, HT29, and SW620. Boyden chambers with 8 μ pores (BD Falcon, Cat. No. 353097) were placed in 24-well cell culture plates. Cells were trypsinized, washed once in DMEM and counted using a hemocytometer. 1 × 10^4^ cells were suspended in 200 μl of serum free media and added to the upper compartment of the Boyden chamber in each well of a 24 well plate. The lower compartment contained 400 μl of complete media with 10% FBS. After incubation for 24 h at 37°C, assays were terminated by scraping the top of the membrane to remove non-migratory cells. The membranes were fixed in 4% paraformaldehyde, stained with crystal violet and mounted on glass slides. Quantification of cells was carried out by counting at least three microscopic fields using a 10X objective. Matrigel coated Boyden chambers (100 μg/ml) were used for the invasion assay. 5 × 10^4^ cells were suspended in 200 μl serum free media and seeded in each well. The lower chamber contained 400 μl of complete media with 10% FBS. After 24 h at 37°C, assays were terminated and cells were quantified as described above for the migration assay. The experiments were repeated at least thrice for obtaining data for statistical significance.

### Gene Expression Analysis by qRT-PCR

Total RNA was isolated from cells using RNeasy Mini Kit (Qiagen, Cat. No. 74104). 1 μg of total RNA was used for cDNA synthesis using iScript cDNA Synthesis Kit (BioRad, Cat. No. 170-8891). Real time quantitative PCR was performed using SYBR Green reagent (SsoFast Eva Green supermix, BioRad, Cat. No. 1725202AP) in a CFX Connect Real Time PCR system (BioRad). Cycling conditions were optimized at initial denaturation for 5 min at 95°C followed by denaturation for 5 s at 95°C and annealing, extension for 45 s at 60°C for 40 cycles. GAPDH was used as the internal control and all CT values were normalized to either internal control or HCT116. Sequences of the primers are listed in Table S1. Three biological replicates were performed for each experiment.

### Antibodies

Primary antibodies against Ki67 was procured from Dako (Cat. No. IR626, Clone MIB1) and caspase 3C from Cell Signaling Technology (Cat. No. 9661). Primary antibodies against β-catenin (Cat. No. 8480), phospho-β-catenin (Cat. No. 9561) were procured from Cell Signaling Technology; E-cadherin (Cat. No. 33-4000), APC (Cat. No. MA1-26185) from Thermo Scientific; α-tubulin (DM1A, Cat. No. T9026) and β-actin (Cat. No. A3853) from Sigma-Aldrich; γ-tubulin (Cat. No. A302-631A) from Bethyl Labs; Pin1 (Cat. No. SC-46660) from Santa Cruz Biotechnology. Anti-mouse and anti-rabbit Alexa-488 conjugated (Cat. No. A11008, A11001) and anti-mouse, anti-rabbit Cy3 conjugated (Cat. No. A10521, A10520) secondary antibodies for immunofluorescence were purchased from Molecular Probes, Invitrogen. Horse Radish Peroxidase (HRP) conjugated anti-mouse (Cat. No. 715-035-150) and anti-rabbit (Cat. No. 711-035-152) secondary antibodies from Jackson Immunoresearch were used for Western Blot analysis.

### Immunofluorescence Staining

Cells were seeded on glass coverslips and allowed to adhere overnight. The media was removed and the cells washed with PBS and fixed in 3.7% paraformaldehyde at room temperature for 5 min. Permeabilization was achieved using PBS containing 1% BSA + 0.5% Triton X-100 for 1–2 min at room temperature. Blocking was performed using 1% BSA + 0.05% Triton X-100 in PBS for 30 min followed by incubation with primary antibody for 1 h. Primary antibodies were used at the following dilutions: α-tubulin – 1:1,000, γ-tubulin – 1:500, β-catenin – 1:200, phospho-β-catenin – 1:200, E-cadherin – 1:200. After washing with PBS, cells were incubated in secondary antibody for 1 h. The cells were washed with PBS and stained with Hoechst 33258 at a dilution of 1:5,000 from a 5 mg/ml stock solution. The cells were again washed with PBS and mounted in Prolong Gold mounting medium (Invitrogen). The slides were allowed to dry overnight before imaging using a Nikon TiE Eclipse epifluorescence microscope.

### Western Blotting

Cells were lysed in 1X Radioimmunoprecipitation Assay (RIPA) buffer containing protease inhibitor (Roche, Cat. No. 05892970001) and phosphatase inhibitor (sodium orthovanadate, New England Biolabs, Cat. No. P0758S) at appropriate concentrations. Laemmli buffer containing β-mercaptoethanol was added and the samples were heated at 95°C for 10 min. Samples were loaded on a 10 or 12% denaturing gel and electrophoresis was performed before transferring the resolved proteins onto PVDF membrane (Millipore). Blocking was done in TBST containing either 5% skimmed milk or 5% BSA followed by incubation in primary antibody overnight at 4°C. Membranes were washed with tris-buffered saline containing 0.1% tween-20 (TBST) and incubated with HRP-conjugated secondary antibody for 2 h at room temperature. Blots were washed extensively with TBST and chromogenic substrate (Luminata Forte, Millipore) was added to develop the chemiluminescent signal. Images were captured in an Image Quant 4000 (GE) gel documentation system. Primary antibodies were used at the following dilutions: β-catenin – 1:1,000, Pin1 – 1:1,000, β-Actin – 1:2,000.

### Drug Sensitivity Assay

Cytotoxicity of bortezomib (ChemShuttle, Cat. No. 179324-69-7) on MBC02 and HCT116 cells was measured using MTT assay. Cells were seeded in quadruplicate in the recommended media in 96 well plates at a density of 3,000 cells per well and allowed to attach overnight at 37°C, 5% CO_2_ and 95% humidity. The cells were incubated for 48 h with different concentrations of bortezomib ranging from 0.03 to 50 nM. Untreated cells were used as control. Following incubation, 20 μl of 5 mg/ml MTT (HiMedia, Cat. No. RM1131-1G) solution was added to the cells and incubated for another 3 h. The media containing MTT was completely removed and the formazan precipitate was dissolved using 100 μl of 1:1 solution of DMSO: methanol. Absorbance was measured at 550 nm with background correction at 655 nm using a microplate reader (iMark, BioRad). The results were analyzed using GraphPad PRISM software (GraphPad, San Diego, CA). The IC_50_ values were calculated and result plotted as the mean ± SEM of the absorbance for each tested dose from three independent experiments. MBC02 cells were also treated with a combination of drugs that are the standard-of-care regimen for treatment of CRC. The therapeutic regimens included 5-fluorouracil (5-FU) + leucovorin (16.78 μg/ml + 391 ng/ml respectively), FOLFOX (oxaliplatin 2.58 μg/ml + 5-FU 16.78 μg/ml + leucovorin 391 ng/ml), FOLFIRI (irinotecan 4 μg/ml + 5-FU 16.78 μg/ml + leucovorin 391 ng/ml), and cetuximab (184 μg/ml) (29). DMSO was used as control for the drug sensitivity test. Experiments were performed in triplicate for statistical significance and results plotted as percent viability for each treatment group.

### Proteasome Activity Measurements

Proteasome 20S activity kit (Sigma-Aldrich, Cat. No. MAK172) was used to measure the proteasome activity of MBC02 and HCT116 cells. 80,000 cells were seeded in each well of a 24 well plate and allowed to adhere overnight. Proteasome substrate stock and proteasome assay loading solution were prepared following the manufacturer's protocol. Media was removed and replaced with a 1:1 ratio of complete media and proteasome assay loading solution and incubated at 37°C, 5% CO_2_ and 95% humidity for 4 h in the dark. Following incubation, the fluorescence intensity was measured at an excitation wavelength (λ_ex_) of 490 nm and an emission wavelength (λ_em_) of 525 nm. For the proteasome inhibition assay, cells were grown to ~85–90% confluency in 10 cm dishes. Bortezomib was used at a concentration of 200 nM to treat cells for different time points ranging from 30 min to 2 h. Untreated cells were used as control in the proteasome inhibition assay.

### Statistical Analysis

All quantitative data are represented as mean ± SEM, calculated from at least three independent biological replicates. Statistical significance was calculated using the Student's *t*-test. *P* < 0.05 was considered statistically significant. GraphPad PRISM (San Diego, CA) software was used for creating all graphs.

## Results

### Primary Tumor Histology

**Figure 1:**
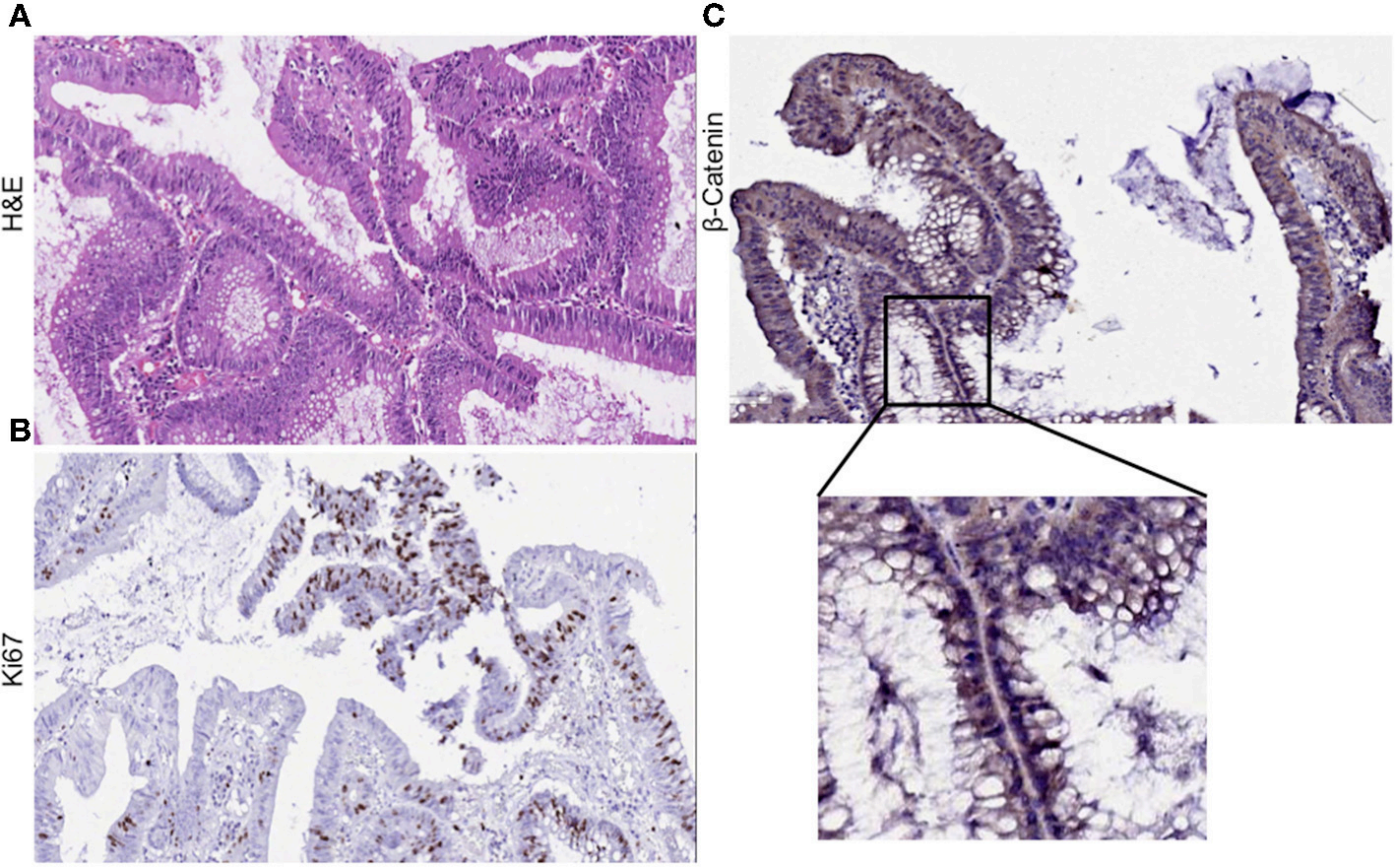
Histological evaluation of primary tumor. **(A)** H&E staining of the primary tumor show moderate differentiation. Cancer cells form irregular glandular structures. **(B)** Ki67 positive staining mark the regions of actively dividing cancer cells within the tumor. **(C)** Primary tumor section was found to express β-catenin abundantly.

Clinical diagnosis of the primary tumor revealed stage IIIA disease (T_2_N_1_M_0_), indicating that the cancer may have progressed into the nearby submucosal tissue and into atleast 3 lymph nodes, although it had not metastasized to distant sites. Histopathological evaluation of hematoxylene-eosin (H&E) stained sections of the primary tumor demonstrated moderate differentiation with cancer cells forming irregular glandular structures. Cancer cells were marked by the dark purple staining of the nuclei (Figure 1A). Strong Ki67 staining in regions of the tumor showed the presence of actively dividing cancer cells (Figure 1B). Upon investigation we also observed an abundant β-catenin expression in the primary tumor (Figure 1C).

### Preliminary Characterization and Cytogenetic Analysis of MBC02

**Figure 2:**
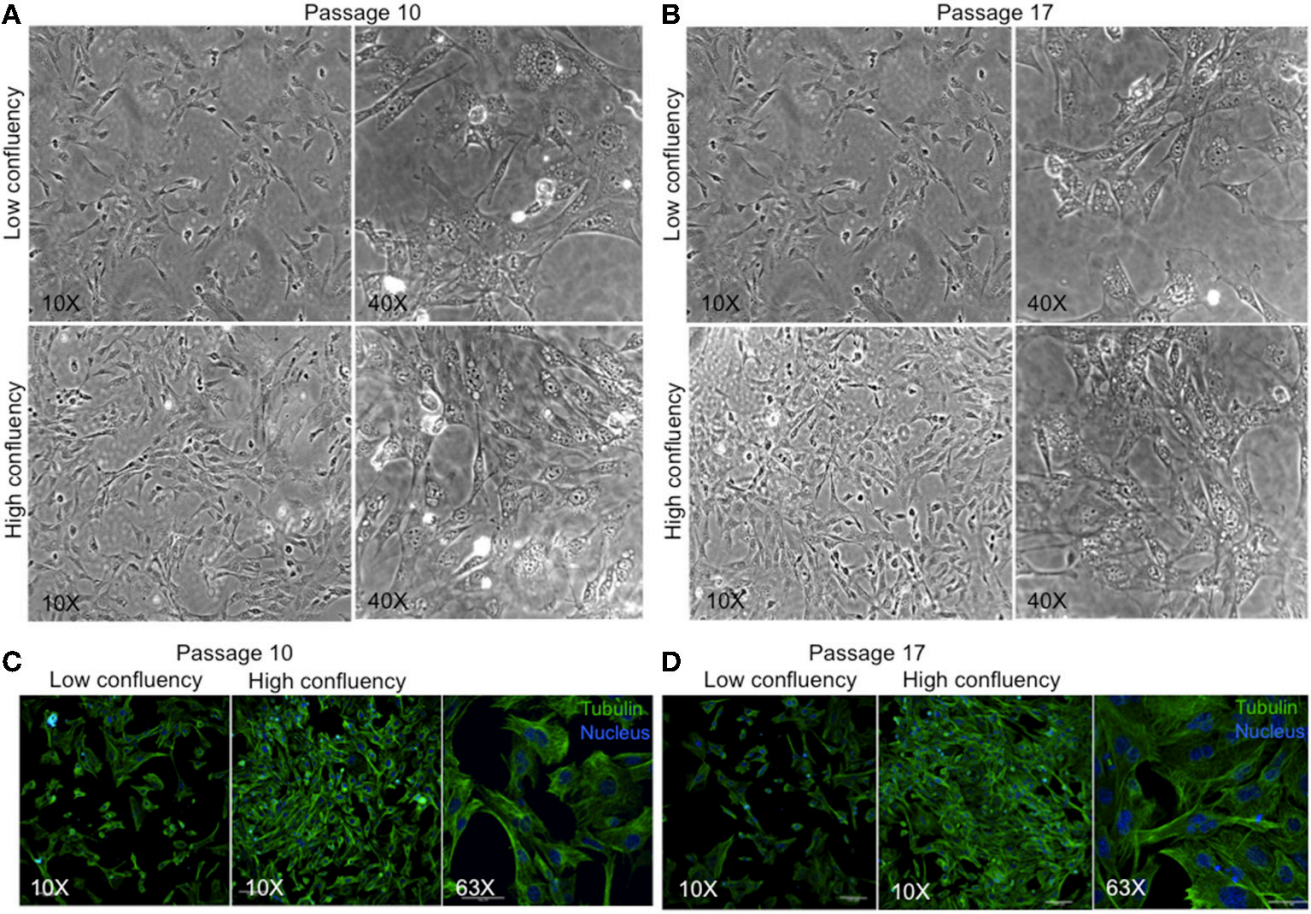
Morphological evaluation of MBC02**. (A,B)** Phase contrast images of passage 10 and 17 of MBC02 at low (10X) and high (40X) magnification show that these cells are adherent to the substratum and have a flattened morphology similar to epithelial cells. **(C,D)** Immunofluorescent staining using anti-α-tubulin antibody show well-formed cytoskeletal network within these cells. The heterogenous nature of MBC02 cell line is apparent due to the presence of some cells that are larger than others.

**Figure 3:**
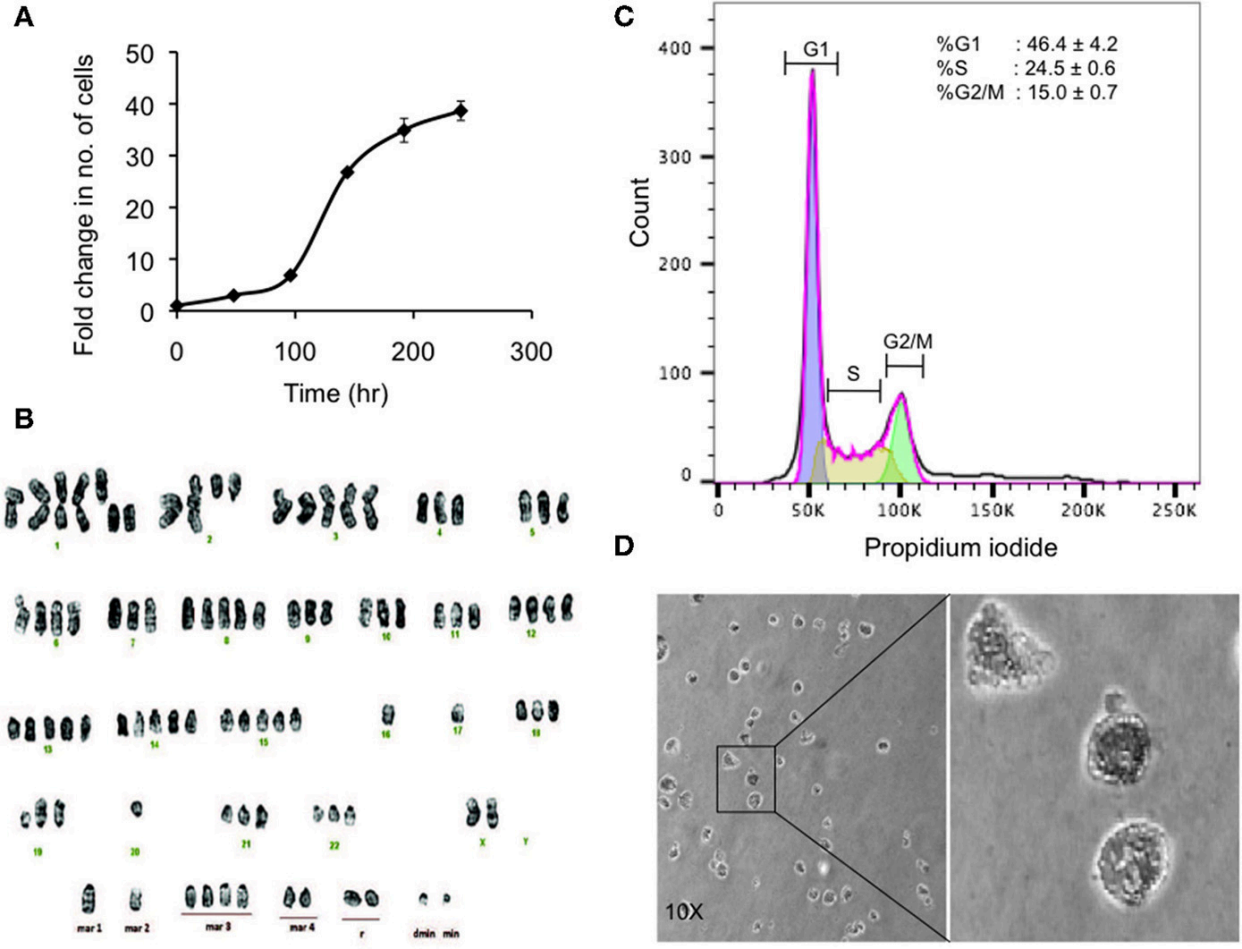
Preliminary characterization of MBC02. **(A)** Population doubling time of MBC02 culture was calculated to be approximately 24 h. **(B)** MBC02 cells exhibit gross cytogenetic defects in the form of both structural and numerical aberrations ranging from centric fission, deletions of either long or short arms of more than one chromosome, duplications and complete loss of an arm of more than one chromosome. The modal chromosome number in MBC02 is 89. **(C)** DNA content analysis of MBC02 cells revealed ~46% cells in the G1 phase whereas, ~24 and ~15%were in S and G2/M phase of the cell cycle **(D)** The ability of MBC02 cells to form colonies in soft agar indicate that these cells can proliferate in an anchorage independent manner and may form tumors if injected in mice.

We have developed a novel cell line, MBC02, from the primary tumor site of a 37-year-old female Indian CRC patient. As controls established colorectal cancer cell lines–HCT116, HT29, and SW620 were used. HCT116 and HT29 were obtained from primary carcinomas of an adult Caucasian male and female patient respectively, whereas, SW620 was derived from a metastatic tumor of a male Caucasian patient^1^. Microscopic examination of passage 10 and 17 under low and high magnification (10X and 40X) revealed that MBC02 cells grew as a monolayer that was strongly adherent to the substratum with a flattened morphology that is characteristic of epithelial cells (Figures 2A,B). Immunofluorescence staining using anti-tubulin antibody further highlighted the epithelial morphology of these cells (Figures 2C,D). Since MBC02 cell line was not derived by clonal selection, the presence of a heterogenous population is apparent with some cells being larger. The average population doubling time for MBC02 was calculated to be approximately 24 h (Figure 3A). In comparison, average population doubling time for HCT116, HT29, and SW620 were calculated to be 20, 25, and 28 h, respectively (Figure S1).

Karyotype analysis of MBC02 cells revealed chromosomal instability in almost all cells. These cells were near-tetraploid with a modal chromosome count of 89. Significant numerical and gross structural abnormalities were observed in chromosomes, some of which were present in multiple copies. A few of the structural rearrangements observed were centric fission detected in one or more copies of chromosome 1, deletion of the short arm of chromosome 1, 2, 4, 5, 11, and 12 and deletion of the long arm of chromosome 2, 9, and 19. In addition, derivative chromosomes as a result of addition of material of unknown origin, probably via translocation, were observed in one or more copies of the long arm of chromosomes 18, 19, 20, 21, and 22 as well as in the short arm of chromosome 19 (Figure 3B). Of note, HCT116 cells are reported to be near-diploid with modal chromosome number of 45 whereas HT29 and SW620 cells are hypertriploid (modal chromosome number of 71) and hyperdiploid (modal chromosomal number of 50), respectively^1^. Cytogenetic analysis of two different passages of MBC02 cells (passage 8 and 16) yielded identical results indicating that the integrity of the cell line is preserved between early and late passages. The abnormal karyotype could have resulted from gross defects in mitosis, both at early and late stages of cell division as well as during cytokinesis. This may lead to the presence of multinucleate cells. Indeed, we observed a ~6-fold increase in the number of cells with multipolar metaphase spindle in MBC02 as compared to HCT116 (Figure S2). We also observed a ~12-fold enhancement in the number of multinucleated cells in asynchronous cultures of MBC02 in comparison to HCT116. In addition, about 15% cells were present in cytokinesis in MBC02 whereas only about 6% of cells were observed to be undergoing cytokinesis in HCT116 (Figure S3). This 2.5-fold increase in the cytokinetic index in MBC02 could be the result of the presence of centrosomal and spindle defects in these cells (Figure S2). Based on the above results, it is apparent that mitotic defects at both early and late stages of cell division may have contributed toward MBC02 cells to have become aneuploid.

DNA content analysis of asynchronous culture of MBC02 cells was performed by measuring the fluorescence intensity of propidium iodide incorporated during the various stages of the cell cycle. We observed that ~46% of cells were at G1 phase representing diploid DNA content (2n). ~24% cells were at S phase (>2n) and ~15% were at the G2/M phase (4n) (Figure 3C). To study the tumorigenic propensity of MBC02 cells, we performed anchorage independent growth assay. We observed the appearance of colonies after 2 weeks of plating the cells. The colonies appeared to be small and rounded (Figure 3D). This observation suggested that MBC02 cells have the potential to form tumors if injected into nude or SCID mice and may prove to be a valuable model system for not only studying cancer pathogenesis but also for screening novel therapeutics for clinical use.

We next examined the original tumor and MBC02 cell line for the presence of common mutations in *KRAS* (codons 12, 13, 61, and 146) and *BRAF* (V600E) that are prevalent among CRC patients. Mutations in these genes were not present in either the tumor or the cell line (Table 1). These molecular markers used for the selection of anti-EGFR therapy were preserved in the patient tumor tissue as well as in the tumor derived primary cell line MBC02.

**Table 1:** Mutational analysis of MBC02.

**CRC sample**	**KRAS mutation**				**BRAF mutation (V600E)**
	**Codon 12**	**Codon 13**	**Codon 61**	**Codon 146**	
Original tumor	WT	WT	WT	WT	WT
MBC02	WT	WT	WT	WT	WT

*MBC02 cells were evaluated for clinically relevant CRC related mutations in KRAS and BRAF. Codons 12, 13, 61, and 146 contained the wild type sequence. The V600E mutation in BRAF was also not present in MBC02 cells*.

### Key Signaling Pathways Are Differentially Regulated in MBC02

CRC pathogenesis is characterized by mis-regulation of a number of molecular pathways that often crosstalk in a complex network. Of note are the Wnt-β-catenin, TGFβ and Notch signaling pathways (30, 31). To investigate whether these pathways are differentially regulated in MBC02 cells in comparison to a standard CRC cell line, HCT116, we quantified the relative mRNA expression of key components of the Wnt-β-catenin, TGFβ and Notch signaling pathways. Our comparative findings revealed that several genes from each of the three pathways were overexpressed in MBC02. In the context of the Wnt-β-catenin pathway, all key signaling molecules showed highly upregulated expression, except for the scaffolding protein Axin1 that showed ~4-fold downregulation as compared to HCT116. A similar trend was observed for the TGFβ pathway with the highest expression of SMAD4 (~3,000-fold). The Notch signaling pathway also showed varied expression levels with Notch4 mRNA being ~23,000-fold higher in MBC02 (Table 2). Our findings present strong evidence that clinically relevant signaling pathways are differentially regulated in MBC02, as compared to HCT116.

**Table 2:** Key signaling pathways are mis-regulated in MBC02.

**Signaling pathway**	**Gene**	**mRNA expression in MBC02**	**Fold change (over HCT116)**
Wnt-β-catenin	Frizzled	Upregulation	1,500
	LRP6	Upregulation	8,000
	Disheveled	Upregulation	4,700
	GSK3b	Upregulation	6,000
	APC	Upregulation	3,650
	Axin1	Downregulation	4
	β-catenin	Upregulation	1,800
TGFβ pathway	SMAD2	Upregulation	1.3
	SMAD 3	Upregulation	3.6
	SMAD 4	Upregulation	3,000
	SMAD 7	Upregulation	13
Notch signaling	Notch1	Downregulation	Undetectable
	Notch2	Upregulation	9,000
	Notch3	Upregulation	500
	Notch4	Upregulation	23,000
	DLL1	Downregulation	Undetectable
	DLL3	Downregulation	Undetectable
	DLL4	Upregulation	2,000
	JAG1	Downregulation	Undetectable
	JAG2	Upregulation	50
	HES4	Upregulation	6
	HEY1	Downregulation	Undetectable

*Differential gene expression in MBC02 cells was determined using qRT-PCR and fold change in mRNA levels was calculated with respect to HCT116. Important signaling pathways that are critical in CRC pathogenesis are mis-regulated in MBC02*.

### MBC02 Cells Lack Cell-Cell Contacts and Are Highly Migratory and Invasive

**Figure 4:**
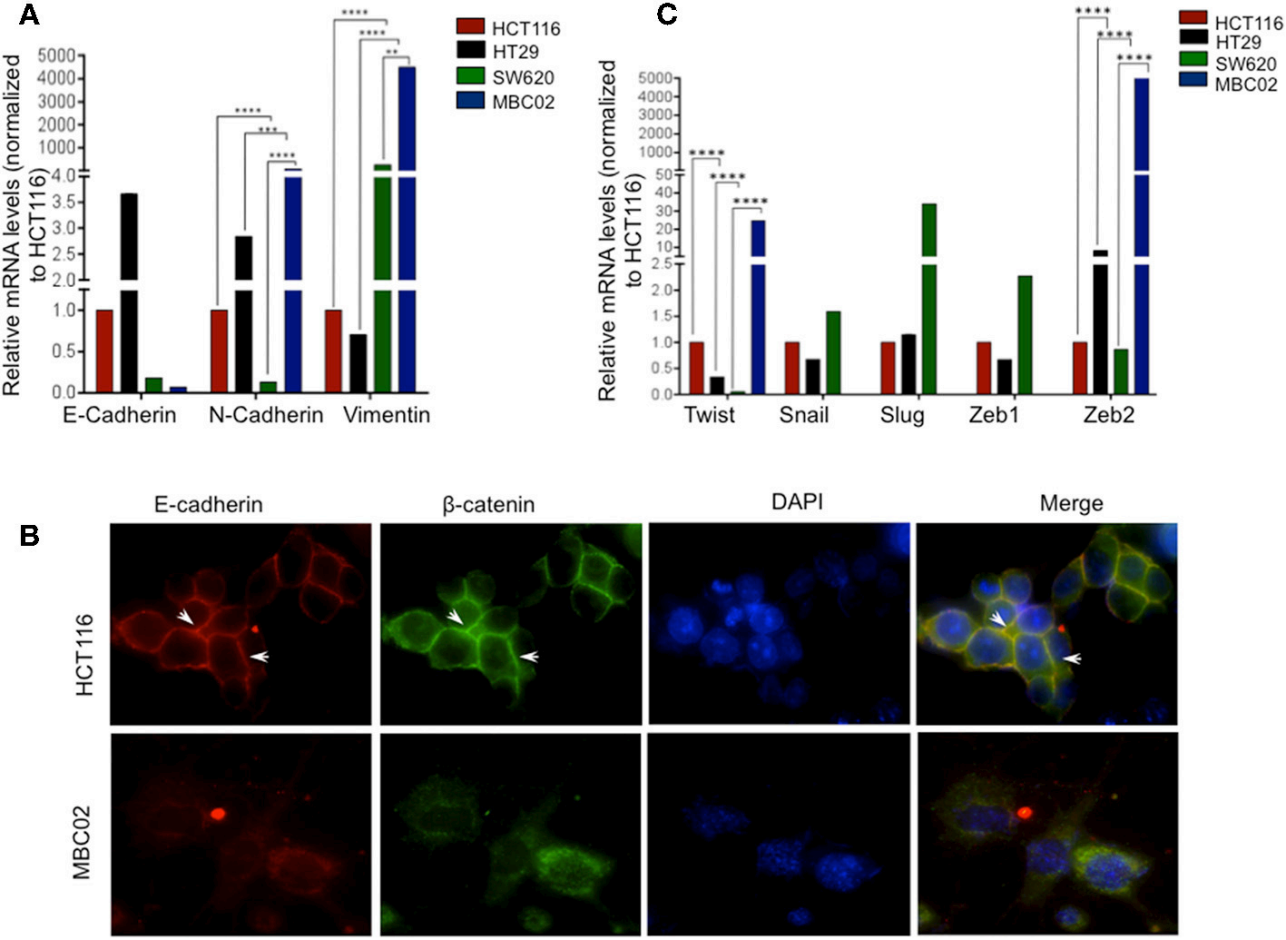
MBC02 exhibits features of epithelial to mesenchymal transition. **(A)** mRNA expression analysis of CRC cell lines using qRT-PCR shows downregulation of E-cadherin and concomitant upregulation of N-cadherin and vimentin, indicating a cadherin switch in MBC02 and SW620 cell lines. **(B)** E-cadherin mediated intercellular junctions are lost in MBC02. Immunofluorescence analysis using anti-E-cadherin and anti-β-catenin antibodies reveal localization of both proteins at the cell-cell junctions in HCT116 (arrowheads), whereas no cortical staining is seen in MBC02. **(C)** EMT related transcription factors Twist and Zeb2 showed increased expression in MBC02 as compared to HCT116. Additionally, Snail, Slug, and Zeb1 are elevated in SW620. *p*-values are ^**^p < 0.01, ^***^p < 0.001, ^****^p < 0.0001.

Altered molecular regulation in a cancer cell resulting in the acquired ability of enhanced cell motility is a key driver in cancer metastasis (9). We set forth to evaluate the relative mRNA expression levels of EMT markers in MBC02 and the established CRC cell lines HCT116, HT29, and SW620. E-cadherin was maximally expressed in HT29 (~3.5-fold higher than HCT116). In SW620, we observed a decrease in E-cadherin expression and almost no detectable mRNA transcript in MBC02. There was a concomitant elevation of N-cadherin and vimentin expression in MBC02 (~6-fold and ~4,000-fold over HCT116). However, there was a more moderate increase in vimentin expression in MBC02 over SW620 (~8-folds) (Figure 4A). The lowered expression of E-cadherin coupled with an enhancement in the levels of N-cadherin and vimentin constitute a typical molecular signature for EMT (32), suggesting that MBC02 may have undergone molecular alterations that could result in its transition from the epithelial to mesenchymal phenotype. Immunofluorescence analysis using an anti-E-cadherin antibody demonstrated localization of E-cadherin at cell-cell junctions in HCT116 as indicated by strong immunofluorescence staining. However, a similar pattern was not seen in MBC02 suggesting a loss of plasma membrane tethered E- cadherin that may have resulted in loss of adherens junction integrity in MBC02 (Figure 4B). Further, we examined the expression of EMT related transcription factors like Twist and Zeb that play critical roles in tumorigenesis and metastasis (33). There was a significant elevation in the levels of Twist (~20-folds) and Zeb2 (~5,000-folds) in MBC02 as compared to HCT116, while Snail, Slug, and Zeb1 transcripts were undetectable. However, all three transcription factors had elevated expression in SW620 as compared to HCT116 (Figure 4C). Together, these molecular alterations in MBC02 could be considered as indicators of these cells having undergone at least a partial EMT.

**Figure 5:**
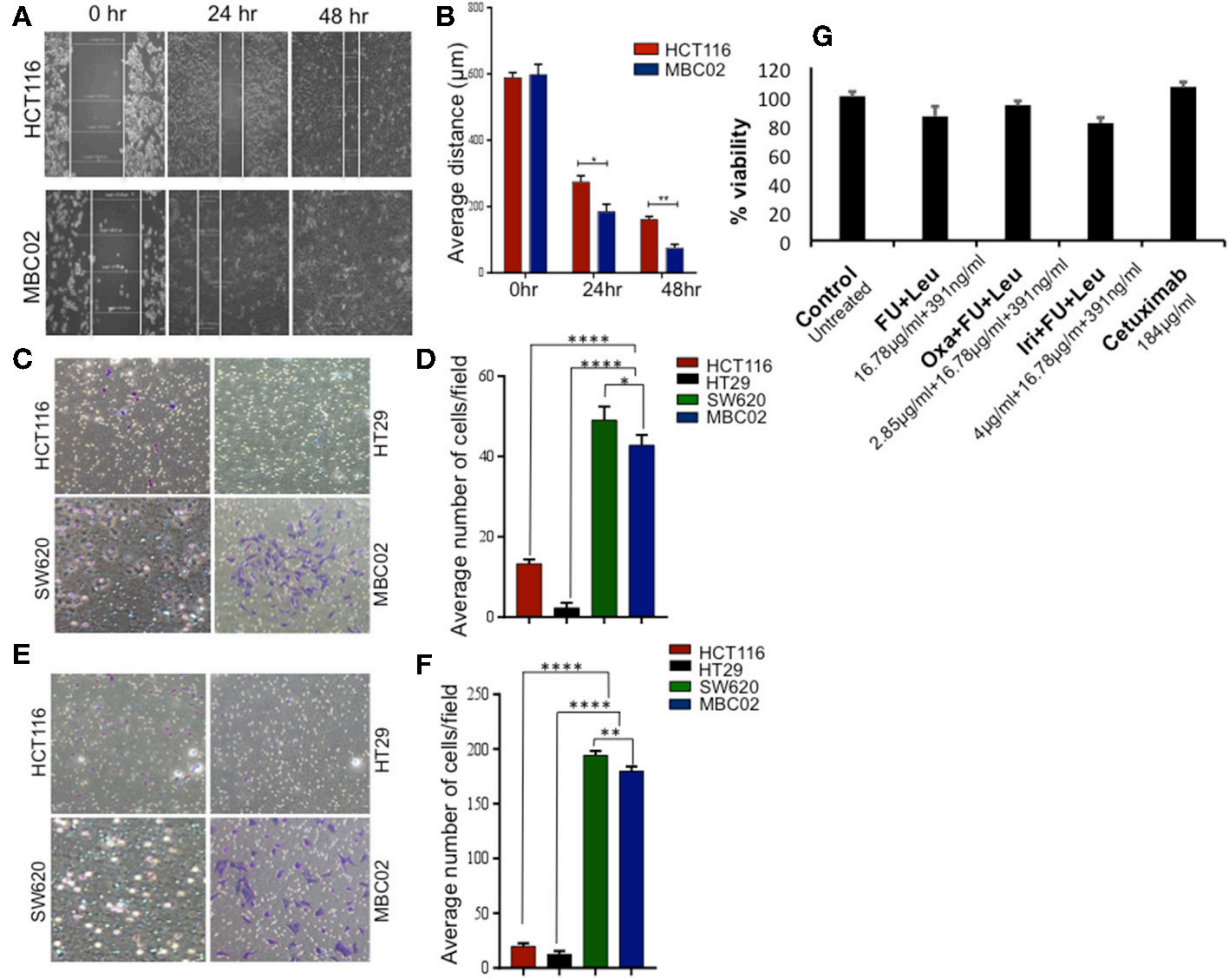
MBC02 is highly migratory and invasive and has attenuated response to standard therapy. **(A,B)** Inherent migratory ability of MBC02 and HCT116 were measured using wound- healing assay. MBC02 cells were able to close the wound within 48 h whereas the wound still persisted in HCT116. **(C,D)** The ability of MBC02 and SW620 cells to migrate through a membrane barrier in a transwell migration assay suggests that MBC02 and SW620 cells have a higher migratory ability as compared to HCT116 and HT29. **(E,F)** Similar results were obtained in the invasion assay where more number of MBC02 and SW620 cells could cross the matrigel coated membrane barrier compared to HCT116 and HT29. **(G)** Clinically relevant drug combination including 5-FU, leucovorin, oxaliplatin, irinotecan and cetuximab were tested for efficacy on MBC02 cells. MBC02 did not show any appreciable response to any of the combinations and was similar to that of control. The data is represented as mean ± SEM calculated from three independent experiments. *p*-values are ^*^p < 0.05, ^**^p < 0.01, ^****^p < 0.0001.

Transition of tumor cells from the epithelial to mesenchymal phenotype endows them with an enhanced capacity for migration. To explore whether there is an enhancement in the motility of MBC02 cells alongside the presence of the EMT signature, we performed a wound-healing assay. After wounding a confluent monolayer of cells, the cells along the wound edge were imaged by microscopy over a 48 h time period. As compared to HCT116, the rate of wound healing was significantly faster in MBC02, which led to more rapid healing of wounded monolayers. HCT116 cells reduced the width of the wound to 185 μm but MBC02 filled the wound in the same time interval (Figures 5A,B). The enhanced motility of MBC02 cells was confirmed by evaluating migration through transwell pores after subjecting the cells to a serum gradient for 24 h. SW620 showed ~3.0-fold higher motility than HCT116, whereas, MBC02 exhibited ~2.5-fold greater motility than HCT116. HT29 cells hardly showed any migratory potential (Figures 5C,D). Similar results were obtained in an invasion assay when these cells were added to matrigel coated Boyden chambers and allowed to migrate across the matrix along a serum gradient. SW620 and MBC02 cells were highly invasive compared to HCT116 (~6.5-fold and ~6-fold higher than HCT116). HT29 did not show any invasive property (Figures 5E,F). These observations indicated that the novel cell line MBC02 is similar to the metastatic cell line, SW620 in the expression pattern of EMT related molecular markers that has resulted in these cells being highly migratory and invasive in nature. Taken together, these results suggest that along with the loss of cell-cell junction integrity, MBC02 cells have gained enhanced migratory ability concomitant with increased invasiveness that present strong evidence of these cells having undergone EMT.

### Response to Standard Therapy Is Attenuated in MBC02

Most often, the treatment regimens for CRC include the nucleoside analog 5-fluorouracil (5-FU) in combination with platinum drugs such as oxaliplatin or topoisomerase I inhibitor irinotecan (34). MBC02 cells were subjected to the various standard treatment regimens for CRC that are currently in clinical practice, namely a combination of 5-FU and leucovorin, FOLFOX (combination of oxaliplatin, 5-FU, and leucovorin), FOLFIRI (combination of oxaliplatin, 5-FU and irinotican) and the epidermal growth factor receptor (EGFR) inhibitor, cetuximab. We found that MBC02 did not show an appreciable response to any of these treatments (Figure 5G, Table S2). EMT is known to induce acquired drug resistance via multiple mechanisms that are not yet well-understood. Upregulation of Twist has been implicated in resistance to 5-FU and oxaliplatin (35, 36). Therefore, activation of EMT not only made MBC02 cells highly migratory and invasive, but also less responsive to standard therapeutic drugs for CRC.

### Expression, Mislocalization, and Cytoplasmic Accumulation of β-Catenin in MBC02

**Figure 6:**
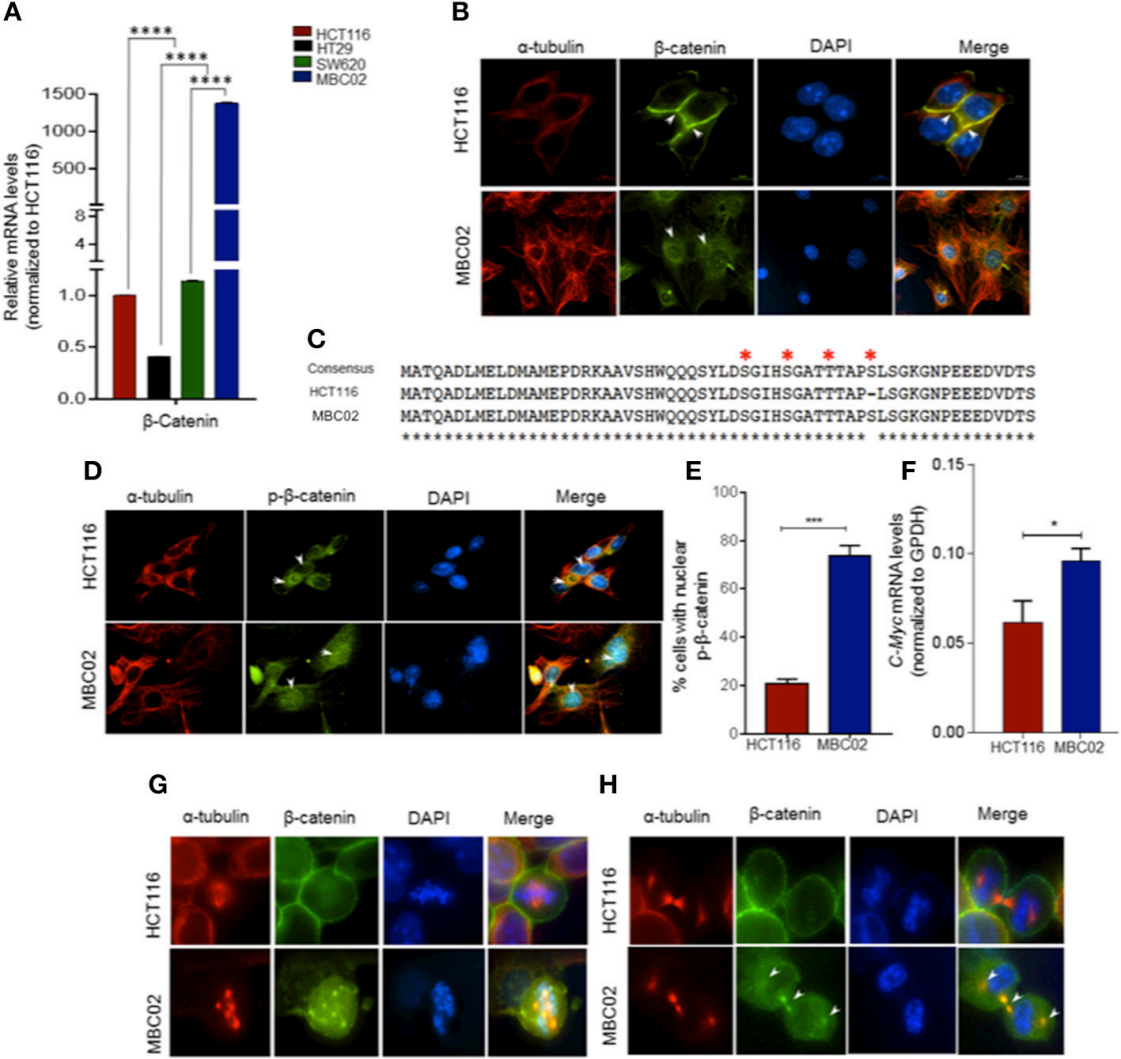
β-catenin is highly activated in MBC02. **(A)** Relative mRNA expression of β-catenin in HCT116, HT29, SW620, and MBC02 cells was measured by RT-PCR and values were normalized to those of HCT116. β-catenin is highly overexpressed in MBC02 as compared to the other cell lines. **(B)** Immunofluorescence imaging of subcellular localization of β-catenin (green) show cortical localization in HCT116, whereas, in MBC02 it shows predominantly cytoplasmic and nuclear distribution. Arrowheads mark β-catenin localized to the cortex and at the inter-cellular junctions in HCT116 and cytoplasmic distribution in MBC02. α-tubulin (red) and nucleus (blue) are also marked. **(C)** Sequencing of MBC02 derived β-catenin shows the N-terminal phosphorylation sites, S33/S37/T41 and S45, contain wild type sequence (red asterisk) whereas in HCT116, there is an in-frame deletion of codon 45. **(D)** Immunofluorescence imaging confirms the presence of phosphorylated β-catenin (green) in MBC02 in the cytoplasm and nucleus (arrows). Phosphorylated β-catenin however, is excluded from the nucleus of HCT116 (arrow). **(E)** Nearly 75% of MBC02 cells show presence of phospho-β-catenin in the nucleus as compared to 20% HCT116 cells. **(F)** β-catenin target gene, *c-Myc* show elevated expression in MBC02 as compared to HCT116. **(G)** Prominent localization of β-catenin (green) at cell cortex is seen in HCT116 during metaphase. There is no visible staining of β-catenin at the spindle poles in HCT116. MBC02 however shows very prominent β-catenin localization at the poles of multipolar spindle during metaphase. **(H)** β-catenin localizes to the intercellular bridge during cytokinesis in MBC02 marked by arrowheads. In HCT116, β-catenin is confined to the cell cortex during early (metaphase) and late (cytokinesis) stages of mitosis, as marked by arrowheads. The data is represented as mean ± SEM calculated from approximately 200 cells per experiment, over three independent experiments. *p*-values are ^*^p < 0.05, ^***^p < 0.001, ^****^indicate statistical significance.

Misregulation of Wnt/β-catenin pathway is a major cause of pathogenesis of CRC and mutations in β-catenin is often used as a marker for disease prognosis (24, 37, 38). We compared the expression levels of β-catenin in MBC02, HCT116, HT29, and SW620 using qRT-PCR analysis. Our results showed a dramatic increase (~1,500-fold) in relative expression of β-catenin mRNA in MBC02 in comparison to all the other cell lines. SW620 however showed similar β-catenin expression as HCT116 (Figure 6A). As the established cell lines have similar expression of β-catenin, we have used HCT116 as the control cell line for all further analyses. Immunofluorescence analysis using anti-β-catenin antibody revealed strong cytoplasmic staining and almost no cortical staining of β-catenin in MBC02 (Figure 6B). In addition, β-catenin also localized to the cytoplasmic microtubules but not to the cortical microtubules in MBC02. In contrast, β-catenin localization at the adherens junctions in HCT116 was confirmed by strong immunofluorescence at the cell-cell contacts (Figure 6B).

The cytosolic accumulation of β-catenin prompted us to investigate the presence of cancer associated stabilizing mutations in MBC02 derived β-catenin. Upon DNA sequence analysis we found that MBC02 contained the wild type sequence at the S33, S37, T41, and S45 phosphorylation sites. In contrast, HCT116 showed the expected in-frame deletion at S45, as reported earlier (34) (Figure 6C). The presence of the wild type phosphorylation sites in β-catenin of MBC02 is indicative of normal phosphorylation. To test this, we immunostained MBC02 and HCT116 cells using a phospho-specific antibody that recognizes β-catenin phosphorylated at S33/S37/T41. We observed significant accumulation of phospho-β-catenin in the cytoplasm and nucleus in MBC02 confirming that phosphorylation of β-catenin remained unimpaired (Figure 6D). Interestingly, a significantly higher fraction, ~75% of MBC02 cells showed nuclear localization of phospho-β-catenin as compared to ~20% for HCT116 (Figure 6E). Upon examination of the mRNA levels of *c-Myc*, a downstream target of β-catenin activation, we observed a ~1.5-fold increase in *c-Myc* expression in MBC02 (Figure 6F). These results suggest that cytoplasmic and nuclear accumulation of β-catenin could partly be responsible for the increased expression of *c-Myc* in MBC02.

Upon investigating the subcellular localization of β-catenin in MBC02 during mitosis, we observed distinct foci of β-catenin at the spindle poles at metaphase that overlapped with α-tubulin (Figure 6G), while during cytokinesis, β-catenin localized prominently at the inter-cellular bridge (Figure 6H). In contrast, the presence of β-catenin at either the spindle poles or at the inter-cellular bridge was not discernible in HCT116 cells, although cortical β-catenin staining was prominent (Figures 6G,H). This differential localization of β-catenin during cell division suggested that the protein may be involved in distinct roles during different phases of the cell division in MBC02.

### Nuclear Retention of β-Catenin Could Have Resulted From Elevated Pin1 Expression in MBC02

**Figure 7:**
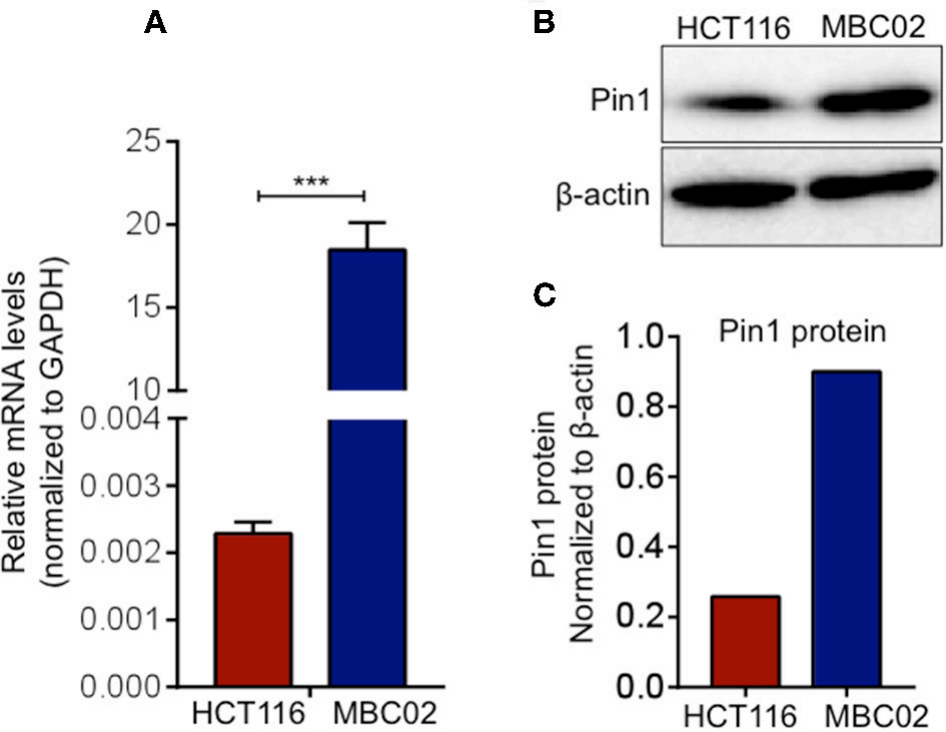
Elevated Pin1 levels in MBC02. **(A)** qRT-PCR measurement of relative mRNA levels of Pin1 in MBC02 and HCT116 cells show that Pin1 transcript levels are highly elevated in MBC02. **(B,C)** Western blot analysis corroborates the RT-PCR data and shows about 3.5 fold higher Pin1 level in MBC02 as compared to HCT116. The data is represented as mean ± SEM calculated from three independent experiments. *p*-values are ^***^p < 0.001.

The prominent presence of cytoplasmic and nuclear β-catenin in MBC02 prompted us to investigate whether alterations in its turnover could result in its enhanced cellular accumulation. Peptidylprolyl Cis/Trans Isomerase, NIMA-interacting 1 (Pin1) is known to cause isomerization induced structural changes in β-catenin that interferes with its interaction with adenomatous polyposis coli (APC) and thus modulate its turnover (39). We examined the level of expression of Pin1 in MBC02 to check whether Pin1 could account for the elevated nuclear accumulation of β-catenin in these cells. Our results demonstrated a dramatic increase of ~9,000-fold in mRNA expression of Pin1 in MBC02 (Figure 7A). Western blot analysis of whole cell lysates of HCT116 and MBC02 showed a significant increase (~3.5-fold) in Pin1 protein levels in MBC02 (Figures 7B,C). In addition, we performed western blot analysis of APC on MBC02 cell lysates to ascertain the presence of APC truncation, which is common in CRC. A band appeared at approximately ~150 KDa size, indicating the presence of a truncated form of APC in MBC02 in comparison to the full-length protein observed in HCT116 (Figure S4). Taken together, the elevated levels of Pin1 and truncated APC in MBC02 could be responsible, at least in part, in retaining β-catenin in the nucleus of MBC02 cells.

### MBC02 Has Elevated Intrinsic Proteasomal Degradation Activity

**Figure 8:**
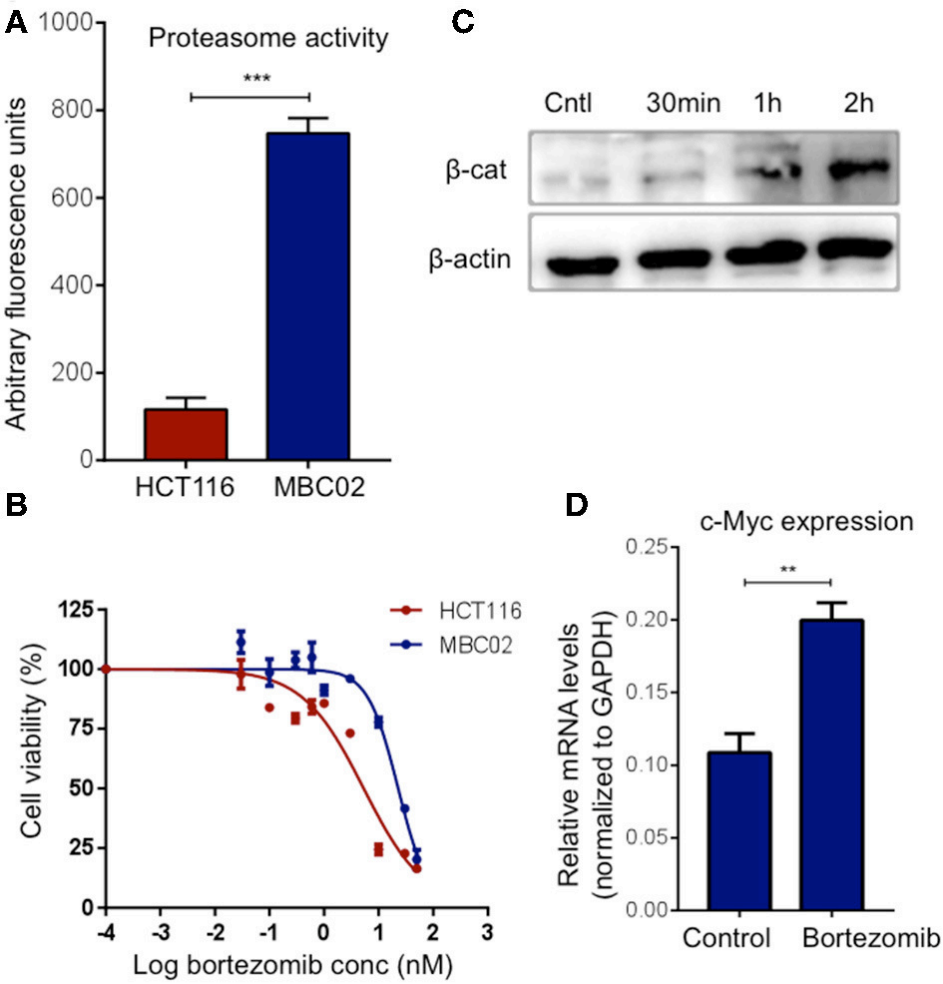
High proteasome activity is observed in MBC02 cells. **(A)** MBC02 showed ~6.5 fold higher endogenous proteasome activity as compared to HCT116. **(B)** MTT assay for checking the cytotoxicity of bortezomib in MBC02 and HCT116 show a higher IC_50_ value for MBC02 (~22 nM) as compared to HCT116 (~5 nM), indicating that MBC02 cells are less sensitive to bortezomib. **(C)** Western blot analysis of cell lysates from bortezomib (200 nM) treated cells shows β-catenin accumulation as early as 30 min post-treatment, thereafter not increasing appreciably up to 2 h. **(D)** Relative transcript levels of downstream target of β-catenin, *c-Myc*, were measured upon treatment with bortezomib (200 nM) and show ~2 fold increase in mRNA levels as compared to untreated control cells. The data is represented as mean ± SEM calculated from three independent experiments. *p*-values are ^**^p < 0.01, ^***^p < 0.001.

Ubiquitin mediated proteasomal degradation of β-catenin is a critical step in the overall regulation of the Wnt/β-catenin signaling cascade and is brought about by the multiprotein complex called the 26S proteasome (22). We postulated that increase in the proteasomal activity could possibly act as a compensatory mechanism for mitigating the high expression of β-catenin in MBC02. Evaluation of basal proteasomal activity in MBC02 revealed an almost 6-fold higher proteasome activity in MBC02 in comparison to HCT116 (Figure 8A). Further, we tested the comparative efficacy of bortezomib, a clinically approved anti-cancer drug that blocks the chymotrypsin activity of the 20S subunit of the proteasome, in MBC02 and HCT116 cells (40–43). A higher IC_50_ value in MBC02 (~22 nM) as compared to that in HCT116 (~5 nM) showed that MBC02 is less sensitive to the proteasome inhibitor compared to HCT116 (Figure 8B). To check whether the inactivation of the proteasome mediated protein degradation system could result in the accumulation of cellular β-catenin, we treated MBC02 cells with 200 nM of bortezomib, retrieved cells at regular time intervals after commencement of treatment and measured the level of β-catenin protein using immunoblotting. Bortezomib treated cells accumulated β-catenin as early as 30 min post treatment and continued thereafter up to 2 h (Figure 8C). We further examined whether the buildup of β-catenin led to alteration in expression of *c-Myc* in these cells. We observed that there was a 2-fold increase in *c-Myc* mRNA levels in treated cells as compared to control (Figure 8D). Our findings suggest that the enhanced proteasomal activity in MBC02 partially compensates for the unusually high expression of β-catenin mRNA. The higher proteasome activity in turn makes MBC02 cells less responsive to the proteasome inhibitor, bortezomib. Further, bortezomib-induced inhibition of proteasome activity allows β-catenin accumulation that brings about transcriptional activation of *c-Myc*.

## Discussion

Cancer cell lines have been the foundation for studies that allow not only elucidation of mechanisms of onset and progression of disease but also contribute toward screening of anticancer molecules for drug development. Patient derived cell lines from cancers originating in specific ethnic groups may potentially be of immense value owing to their inherent population specific genetic variations that ultimately influence disease progression. Although CRC cases are on the rise in India, there is a dearth of Indian patient derived cell lines that can facilitate studies to unravel the characteristics of CRC pathogenesis in the Indian population by serving as a relevant model to study various aspects of disease progression and its subsequent management in the clinic. Intra-tumor heterogeneity (ITH) or the coexistence of genetically distinct sub-clonal populations of cells within the same tumor, is the most defining aspect of all cancers as it influences its response to a given therapy. Conventional cell line models fail to capture this heterogeneity of tumors as they are mostly clonal and highly homogenous in nature. In this study, we have established a primary cancer cell line, which is derived from a CRC patient addressing the above problem by propagating polyclonal tumor cells closely resembling the native patient tumor.

We observed morphological differences between MBC02 and the widely used established CRC cell lines (Figure 2). Gross numerical and structural anomalies in more than one chromosome were noted, indicating global chromosomal instability (CIN) in MBC02, a feature that is common in 80–85% of all CRC cases (44) (Figure 3B). The underlying reason could be the presence of multipolar metaphase spindles arising from the supernumerary centrosomes that ultimately result in defective mitosis and cytokinesis in these cells leading to the formation of large number of multinucleate cells (Figures S2, S3). Tumorigenicity of a cell line upon implantation in mice serves as an invaluable tool for testing and developing novel therapeutics. The ability of MBC02 cells to form colonies in soft agar in an anchorage independent manner (Figure 3D) alludes to its potential to be developed as a colorectal cancer model. The absence of the clinically relevant mutations in *KRAS* and *BRAF* (Table 1) in MBC02 suggests that cancer pathogenesis may have been driven by some other mechanism. Pathogenesis and progression of CRC have been associated with mis-regulation of inter-connected signaling pathways, of which Wnt-β-catenin, TGFβ, and Notch are prominent (12, 45, 46). These have also been implicated in the activation of the EMT program leading to metastasis, resistance to chemotherapy and poor survival (12). Our in-depth analysis of mRNA expression of Wnt-β-catenin, TGFβ, and Notch pathways highlight the presence of differentially regulated gene expression patterns in MBC02 (Table 2). Aoki et al. (47) demonstrated that activation of β-catenin/TCF contributed to chromosomal instability (CIN) in many cancers including gastrointestinal malignancies, which is independent of the p53 status. Therefore, taken together, these molecular features sets MBC02 apart from the widely used established CRC cell lines.

Under normal physiological conditions, an optimal level of E-cadherin expression is necessary to maintain structural integrity of the tissue via cell-cell adherens junction formation. In MBC02, there is a switch from E-cadherin to N-cadherin expression, accompanied by gain of expression of vimentin, along with upregulation of Twist and Zeb2 (Figures 4A,C). These transcription factors have been reported to bind to the E-cadherin promoter and bring about its transcriptional repression (48). Also, a positive correlation has been established between Twist expression, EMT and poor clinical prognosis (49). Therefore, it is reasonable to assume that high levels of these transcription factors may have resulted in reduction of E-cadherin mRNA expression in MBC02. Moreover, there is loss of E-cadherin from the cell-cell junctions in MBC02 (Figure 2B). In a tumor contexture, the functional outcome of this could be the acquired ability of the cancer cells to break off from the tumor mass and metastasize. MBC02 cells do exhibit an enhanced migratory and invasive nature that is similar to that of the established metastatic CRC cell line SW620 (Figures 5A–F) Therefore, our results demonstrate that the EMT program is likely to be activated in MBC02.

Recent years have seen EMT emerge as one of the major determinants of response to chemotherapy with clear correlation between the EMT phenotype and diminished efficacy of chemotherapeutic agents (50). The standard-of-care treatment regimen for CRC consisting either of a combination of 5-FU and leucovorin, FOLFOX, FOLFIRI or the targeted therapy cetuximab did not have any appreciable effect on these cells (Figure 5G). Although the mechanisms governing EMT induced drug resistance are not well-understood, several molecules are implicated to have key contributions, including EMT transcription factors Twist and Zeb (51). In addition to transcriptional repression of E-cadherin, these molecules also influence drug response (11, 52, 53). Low Twist and Zeb expression have shown favorable treatment outcome in patients and therefore, these could be valuable indicators for predicting drug response (54).

Membrane tethered E-cadherin anchors β-catenin at the plasma membrane forming a cadherin-catenin complex that not only is a component of functional adherens junctions but also shields β-catenin from proteasomal degradation (19, 20). Loss of E-cadherin in MBC02 (Figure 4B) may have resulted in compromised cell-cell junctions, releasing β-catenin into the cytoplasm, hence increasing the cytoplasmic pool of free β-catenin (Figure 6B). In MBC02, the presence of conserved, wild type residues at the regulatory site of β-catenin (Figure 6C), as well as immunofluorescence analysis using phospho-specific antibody demonstrate that phosphorylated β-catenin is present in abundance in both cytosolic and nuclear compartments (Figures 6D,E). This observation is supported by the work that showed that N-terminally phosphorylated β-catenin does not associate with cadherins and is mainly cytosolic and that T41/S45 phosphorylated β-catenin is largely nuclear (55). Wild type S45 along with S33/S37/T41 ensures efficient phosphorylation that localizes phospho-β-catenin to the nucleus, as confirmed by immunofluorescence analysis of MBC02. HCT116 however, contains a mutation at the S45 site (ΔS45), precluding phosphorylation at that site and excluding β-catenin from localizing to the nucleus (Figures 6D,E). The cytoplasmically accumulated β-catenin eventually translocates into the nucleus to bring about transcriptional activation of target genes as seen by the elevated expression of *c-Myc* in MBC02 (Figure 6F).

Our observation that β-catenin is present at the centrosomes in MBC02 (Figures 6G,H) is supported by earlier studies that have brought forth a relatively novel function of β-catenin in maintaining centrosomal integrity. Further, overexpression of β-catenin has been reported to result in disorganized centrosomes and loss of centrosomal cohesion leading to spindle organization defects (56, 57). The massive upregulation of β-catenin in MBC02 could be responsible for its prominent localization at the spindle poles and the cytokinetic bridge (Figures 6G,H). Although localization of β-catenin to the inter-cellular bridge during late telophase has been reported earlier, its function in cytokinesis or its role in inducing cytokinetic defects has not been documented (57). It may be speculated that the increased amount of β-catenin within a localized area may perhaps play a role in inducing cytokinetic defects that may eventually lead to increase in the overall number of multinucleate cells in MBC02 (Figure S3).

Malignant transformations often lead to changes in the regulation of β-catenin turnover and result in abnormal accumulation in subcellular compartments (26, 27). Apart from being a core component of the destruction complex, the protein APC plays a crucial role in nuclear export of β-catenin. Phosphorylation dependent binding of Pin1 to β-catenin at the pS247P (phospho-Serine 247-Proline 248) motif brings about structural isomerization of β-catenin and prevents binding of APC to β-catenin. Pin1 upregulation has been reported in CRC and has been positively correlated with β-catenin expression (58–62). Most cancer-related mutations in APC occur within the Mutation Cluster Region (MCR) of APC that produces a N-terminal trunctated protein (~150 KDa, instead of the full-length 310 KDa protein) which is unable to bind β-catenin and mark it for ubiquitination and subsequent proteasomal degradation (50). The elevated Pin1 levels in MBC02 (Figures 7A–C), together with truncated APC (Figure S4) could have a negative influence on the binding of β-catenin to APC, thereby inhibiting its nuclear export resulting in the accumulation of β-catenin in the nucleus (Figure 6D).

The ubiquitin-proteasome machinery plays an important role in maintaining cellular homeostasis by regulating the constant flux of synthesis, degradation and re-synthesis of proteins in the cellular milieu. Cancer cells have evolved various mechanisms to counter the abnormal production of proteins—altered proteasomal activity is one such mechanism (63, 64). MBC02 has approximately 6-fold higher basal proteasome activity over HCT116 (Figure 8A) and bortezomib mediated blocking of the proteasome led to a rapid accumulation of β-catenin (Figure 8C). However, it is unclear whether the high intrinsic proteasomal activity in MBC02 is a result of the dramatic transcriptional upregulation of β-catenin or the increased β-catenin mRNA level is a countermeasure for the elevated proteasomal activity in this cell line.

Proteasomal degradation of N-terminally phosphorylated β-catenin is well-documented (26, 27). Recently, it has been established that phosphorylation of β-catenin at S33/S37/T41 can also be achieved by NIMA-related protein kinase 2 (Nek2) and that binding of Nek2 to β-catenin prevents interaction of β-catenin with the E3 ligase β-TrCP resulting in inhibition of ubiquitination and proteasomal degradation (65). However, it is yet to be established whether Nek2 mediated phosphorylation of β-catenin is a mechanism that could shield β-catenin from GSK3β mediated phosphorylation and proteolytic degradation. It has been proposed that Nek2 mediated phosphorylation may be an alternate regulatory mechanism that could influence β-catenin stability that is independent of GSK3β (66). For our immunofluorescence analysis, we have used a phospho-S33/S37/T41 specific antibody that is unable to distinguish between the GSK3β or Nek2 mediated phosphorylated forms. We therefore speculate that in addition to the GSK3β mediated phospho-β-catenin, there may be a significant pool of Nek2 mediated phospho-β-catenin present that may escape proteasomal degradation and hence accumulate in the cytoplasm in MBC02.

Recent advances focusing on dissecting the molecular dynamics that govern cancer onset and progression are based on studies originating in the western countries with little or no data on Indian patients. India is known to be a genetically diverse population (4), therefore it may be reasonable to speculate that there may be differences in the underlying mechanisms of disease onset. Small molecular perturbations at the genetic level may translate to more drastic mechanistic differences, which could result in currently available therapies being rendered suboptimal in treating patients from various populations. Such molecular differences have the potential to be harnessed for developing either as molecular markers or targeted therapeutics that could be more effective in certain populations. Comparison of MBC02 cell line, originating from Indian patient, with the established Caucasian cell lines such as HCT116, HT29 and SW620, highlight such differences that may indicate underlying mechanistic differences in pathogenesis of CRC in these populations. However, detailed analyses from large patient cohorts would be needed in order to validate these findings that could be further utilized to design specific drugs tailored to targeted patient groups.

## Author Contributions

SM characterized MBC02, performed the cell-based assays, molecular characterization, high-resolution fluorescence imaging, and wrote the manuscript. HK performed the DNA sequencing and Western blot analysis. SK performed the MTT assays for determining the IC50 values. LS performed the anchorage independent growth assay. MJ performed the combinatorial drug sensitivity assay. AB developed the primary cell line along with initial characterization. MB performed immuno-histochemical analysis and reporting for the original colorectal tumor tissue. SVSM critically reviewed the manuscript and gave technical inputs on experimental design. MR supervised the design and execution of the experiments and data analysis. MR and AD supervised the overall study.

### Conflict of Interest Statement

SM, SK, and MR are employees of Invictus Oncology Pvt. Ltd., and SM and MR hold equity of IOPL. The remaining authors declare that the research was conducted in the absence of any commercial or financial relationships that could be construed as a potential conflict of interest.

## Footnotes

^1^https://www.atcc.org
