# Supplementary material for: Activation of Epithelial-Mesenchymal Transition and Altered β-Catenin Signaling in a Novel Indian Colorectal Carcinoma Cell Line
Source: Front Oncol. 2019 Feb 15;9:54. doi: 10.3389/fonc.2019.00054 (PMC6385509; doi:10.3389/fonc.2019.00054)
Supplement: Supplementary file 1 [file Data_Sheet_1.docx]

***Supporting information:***

**Activation of epithelial-mesenchymal transition and altered β-catenin signaling in a novel Indian colorectal carcinoma cell line**

**Sanghamitra Mylavarapu^1,2^, Harsh Kumar^3,4^, Smita Kumari^1^, L. S. Sravanthi^5^,** **Misti Jain^6^, Aninda Basu^6^, Manjusha Biswas^6^, Sivaram V S Mylavarapu^3,4^, Asmita Das^2^ and Monideepa Roy^1,5^***

**Supplementary figures:**

**
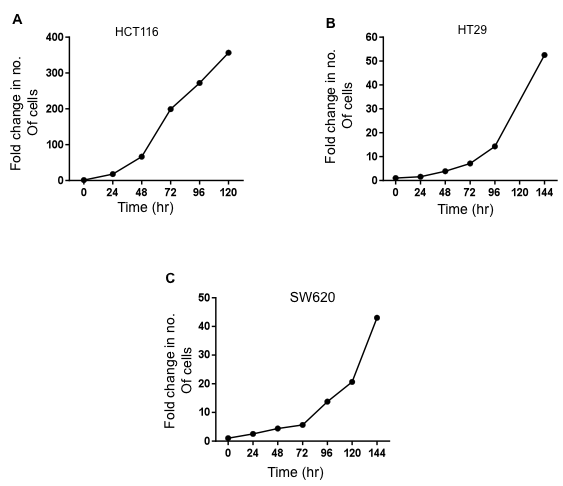
**

**Supplementary figure S1: Population doubling time of established CRC cell lines.**

We have used HCT116, HT29, SW620 as controls for our studies. The average population doubling time for HCT116, HT29 and SW620 were calculated to be 20, 25 and 28 hours respectively.

**
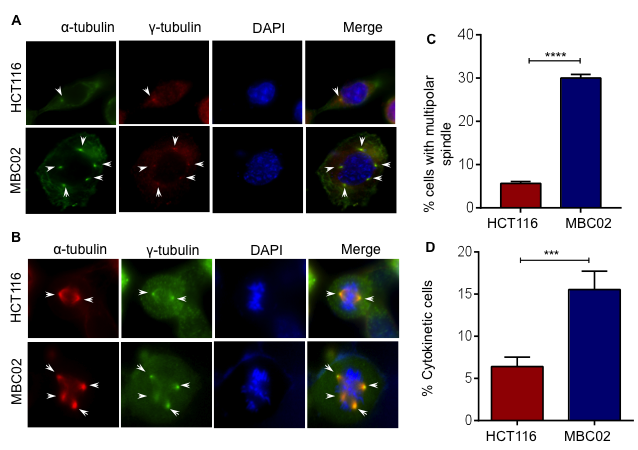
**

**Supplementary figure S2: Supernumerary centrosomes and spindle organization defects in MBC02.**

**(A):** Immunofluorescence staining with anti-α-tubulin and anti-γ-tubulin antibodies revealed multiple centrosomes in MBC02 at interphase (arrowheads). **(B):** Multipolar spindle apparatus, marked by arrowheads, observed at metaphase in MBC02 but not on HCT116 cells. Arrowheads mark spindle poles. **(C):** 30% of MBC02 cells show multipolar spindles in comparison to 5% of HCT116 cells. Approximately 100 metaphase cells per experiment were counted to calculate the percentage of cells exhibiting multipolar spindle. **(D):** Similar numbers of cells undergoing cytokinesis were counted to calculate the percentage of cytokinetic cells. Significantly higher numbers of cells (~ 2.5 fold) were arrested at cytokinesis in MBC02 as compared to HCT116.The data is represented as mean ± SEM calculated over three independent experiments. p values are *p < 0.05, **p < 0.01, ***p < 0.001, ****p < 0.0001.

**
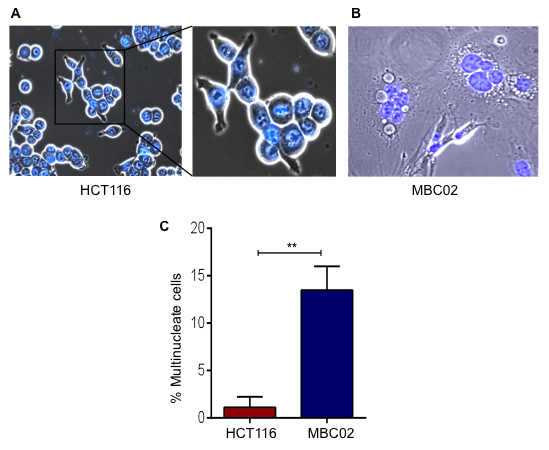
**

**Supplementary figure S3: MBC02 cells show a prevalence of multinucleated cells and are cytokinetically arrested.**

**(A, B):** Brightfield images of HCT116 and MBC02 cells show the presence of many multinucleate cells, especially in MBC02 (~12 fold higher). **(C):** Live cells were stained with Hoechst 33258 and imaged. At least 100 cells per experiment were counted in asynchronous cultures of HCT116 and MBC02 to calculate the percentage of cells exhibiting multiple nuclei. The data is represented as mean ± SEM calculated over three independent experiments. *p < 0.05, **p < 0.01, ***p < 0.001.

**
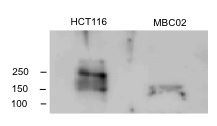
**

**Supplementary figure S4: APC truncation in MBC02.**

We made whole cell extracts from HCT116 and MBC02 cells and separated them by gel electrophoresis followed by Western blotting. Blots were probed with anti-APC antibody. A truncated band of ~150 KDa was observed in MBC02 cell line indicating that APC is mutated. HCT116, on the other hand, showed multiple bands including the one corresponding to full length APC (~310 KDa).

**Table S1:** Primer sequences used for qRT-PCR evaluation of mRNA expression of signaling pathway components in MBC02 and HCT116 cells

| **Gene** | **Forward** | **Reverse** |
| --- | --- | --- |
| GAPDH | 5' TCTGACTTCAACAGCGACAC 3' | 5' TACTCCTTGGAGGCCATGT 3' |
| E-cadherin | 5' CCAAAGACAGAGCGGAACTA 3' | 5' GAGACTCCTCCATTCCTTCC 3' |
| N-cadherin | 5' TTGTGGTGGGAGCAGTAAGT 3' | 5' CCAATCTCATGGTCTCATCC 3' |
| Vimentin | 5' ACTCCCTCTGGTTGATACCC 3' | 5' ATTGCTGCACTGAGTGTGTG 3' |
| Twist | 5' ATGCATTCTCAAGAGGTCGT 3' | 5' GGCCAGTTTGATCCCAGTAT 3' |
| Snail | 5' CGGGCAATTTAACAATGTCT 3' | 5' AAATGTAAACATCTTCCTCCCA 3' |
| Slug | 5' ATGCATTTCTTCACTCCGAA 3' | 5' CATGAATTCCATGCTCTTGC 3' |
| ZEB1 | 5' GAAATCCTCTCGAATGAGCA 3' | 5' TCCTGCAATTCTTCCATCTC 3' |
| ZEB2 | 5' GTTCGAGGAGGAAGAGGAAG 3' | 5' TGGTCTGATTTGGTTTCCAT 3' |
| IQGAP1 | 5' CGCTGCTCTGAACTCTAAGG 3' | 5' TCCTTTCATTTCCCTAGGCT 3' |
| Rac1 | 5' TCCTGTAGTCGCTTTGCCTA 3' | 5' TGTTGTAGTGGCTGAAGGGT 3' |
| CDC42 | 5' GCATATGAGGAACCCTAGAGC 3' | 5' AATTGGAAACTGCAACCAAA 3' |
| CLIP-170 | 5' TCTCCACGACACAGAGGATT 3' | 5' CAGAAGGTTTCGTCGTCATT 3' |
| EB1 | 5' TTTCTGGTGGTAGCTTGTCC 3' | 5' CTGTCATTTGAACAATGGCT 3' |
| Fzd | 5' GCTGCAAGAGCTACGCTATC 3' | 5' GGACCAGATCCAGAAGCC 3' |
| LRP5 | 5' ATCAAACAGCAGTGCGACTC 3' | 5' GAGAGGATGATGCCAATGAC 3' |
| LRP6 | 5' TCTCTTCCAGGAATGTCTCG 3' | 5' GGGAAGTAAGTGCCTTTGGT 3' |
| DVL2 | 5' GGGAAGTAAGTGCCTTTGGT 3' | 5' CTTCCTCAGGCTGCTGTCTA 3' |
| GSK3b | 5' TGCACTCTTCAACTTCACCA 3' | 5' TGTCCACGGTCTCCAGTATT 3' |
| APC | 5' GCTATTCAGGAAGGTGCAAA 3' | 5' GATGAAATGGTGATCCCAGA 3' |
| Axin1 | 5' CGTGTGCTGGGATCTACTTT3' | 5' AAGCTGTGTTGAAGGCACTC 3' |
| b Catenin | 5' CAATGGCTTGGAATGAGACT 3' | 5' CCCATCTCATGTTCCATCA 3' |
| SMAD2 | 5' AAACTGAGTGTCCCAAAGGTT 3' | 5' GGTTTGCCTAGATCAAGAAGC 3' |
| SMAD3 | 5' AGTGCTGGTGACTGGATAGC 3' | 5' AGACAAGGATCTGTGTCCCA 3' |
| SMAD4 | 5' GGTCAGGTGCCTTAGTGACCA 3' | 5' CTGACGCAAATCAAAGACCT 3' |
| SMAD7 | 5' ACGCTGTTGGTACACAAGGT 3' | 5' AGCTGATCTGCACGGTAAAG 3' |
| NOTCH1 | 5' AGTTTGGGAGGAGCAGATTT 3' | 5' GCTGAGCCAAGTCTGACG 3' |
| NOTCH2 | 5' GGGTTTCACTGGATCCTTCT 3' | 5' AGATTCACCAGGGTCTGACA 3' |
| NOTCH3 | 5' AGGACATGCAGGATAGCAAG 3' | 5' AGTCTCTCCTGGGCTACGTC 3' |
| NOTCH4 | 5' CCAGAGCAGACATCTTCCAC 3' | 5' TGCAATTCTTGGTTCCAACT 3' |
| DLL1 | 5' CAGGTTCTCCTCCTGAGGTC 3' | 5' GCGTAATTCAGTTCACCCAT 3' |
| DLL3 | 5' TCCCTACCCTTCCTCGATT 3' | 5' GAAGATGGCAGGTAGCTCAA 3' |
| DLL4 | 5' ACACCTTTGGGTGTCTGTCT 3' | 5' GCTACTGCCACTCTCTCTGG 3' |
| JAG1 | 5' CAGAGCTTAAACCGAATGGA 3' | 5' GGATTCTAAGTCAGCAACGG 3' |
| JAG2 | 5' CTTGTTCTTTCGGTGCTGTC 3' | 5' GACTCAACAGAACCGTCTCG 3' |
| HES4 | 5' CACTCGAAGCTGGAGAAGG 3' | 5' GCCAGACACTCGTGGAAG 3' |
| HEY1 | 5' TGCCTCCTATAGCAGAAAGG 3' | 5' CCAACACTCCAAATGAGACC 3' |
| c-Myc | 5’ CAGCTACGGAACTCTTGTGC 3’ | 5’ CAAGACTCAGCCAAGGTTGT 3’ |
| Pin1 | 5’ TGCCTTCAGCAGAGGTCAGA 3’ | 3’ ACAATTCGGCCCTCGAGTCT 3’ |

**Table S2:** IC_50_ values of compounds on HCT116, SW620 and MBC02 cell lines

| **IC_50_ (µM)** | **HCT116** | **SW620** | **MBC02** |
| --- | --- | --- | --- |
| **Oxaliplatin** | 6.2±0.82μM | 3.7±1.3μM | 6.6±1.9μM |
| **Irinotecan** | 0.35±0.072μM | 5.41±1.5μM | 1.128±0.04μM |
| **Cetuximab** | 392μM | 495μM | IC_50_ value not reached |
| **5-Fluorouracil** | 2.7μM | 25.7μM | - |
